# Drug Delivery Technology Development in Canada

**DOI:** 10.3390/pharmaceutics11100541

**Published:** 2019-10-17

**Authors:** Kishor M. Wasan, Ildiko Badea

**Affiliations:** 1 College of Pharmacy and Nutrition, University of Saskatchewan, Saskatoon, SK S7N 2Z4, Canada; 2 Faculty of Pharmaceutical Sciences, University of British Columbia, Vancouver, BC V6T 1Z3, Canada

**Keywords:** drug delivery, pharmaceutics, drug development, formulation and dosage form development, translational research, biologicals, small molecules, clinical trials, pharmacokinetics, medical devices, route of administration

## Abstract

Canada has a long and rich history of ground-breaking research in drug delivery within academic institutions, pharmaceutical industry and the biotechnology community. Drug delivery refers to approaches, formulations, technologies, and systems for transporting a pharmaceutical compound in the body as needed to safely achieve its desired therapeutic effect. It may involve rational site-targeting, or facilitating systemic pharmacokinetics; in any case, it is typically concerned with both quantity and duration of the presence of the drug in the body. Drug delivery is often approached through a drug’s chemical formulation, medical devices or drug-device combination products. Drug delivery is a concept heavily integrated with dosage form development and selection of route of administration; the latter sometimes even being considered part of the definition. Drug delivery technologies modify drug release profile, absorption, distribution and elimination for the benefit of improving product efficacy and safety, as well as patient convenience and adherence. Over the past 30 years, numerous Canadian-based biotechnology companies have been formed stemming from the inventions conceived and developed within academic institutions. Many have led to the development of important drug delivery products that have enhanced the landscape of drug therapy in the treatment of cancer to infectious diseases. This Special Issue serves to highlight the progress of drug delivery within Canada. We invited articles on all aspects of drug delivery sciences from pre-clinical formulation development to human clinical trials that bring to light the world-class research currently undertaken in Canada for this Special Issue.

This special issue in *Pharmaceutics,* entitled “*Drug Delivery Technology Development in Canada*” was put together to highlight the outstanding achievements and international impact of Canadian scientists in the field of drug delivery. For over 30 years Canadian scientists from leading Canadian research-intense academic institutions, pharmaceutical industry and the biotechnology community have played a vital role in the development of and implementation of novel drug delivery technologies that have made an impact on a number of diseases from cancer to infectious diseases.

Drug delivery encompasses a spectrum of approaches, formulations, technologies, and systems for carrying active pharmaceutical ingredients into the body. The main focus is to achieve optimal pharmacokinetic profile, often attained by active targeting. To achieve this goal, the drugs are formulated in chemical drug delivery systems, incorporated in devices or combination of these two strategies [1]. Drug delivery technologies modify drug release profile, absorption, distribution and elimination for the benefit of improving product efficacy and safety, as well as patient convenience and compliance [2]. 

This Special Issue on Drug Delivery Technologies in Canada highlights the progress of drug delivery research and development within Canada. We invited articles on all aspects of drug delivery sciences from pre-clinical formulation development to human clinical trials that bring to light the world-class research currently undertaken in Canada. In the next paragraphs we summarize the contributions to our special issue. 

Babu V.Sajesh et al. [3] discusses the limitations faced by therapeutic agents to reach their target in the brain by crossing the blood-brain barrier by using HAV6, a cadherin binding peptide, the blood-brain barrier was opened transiently, leading to improvement of the delivery of a therapeutic agent in a murine brain tumour model. This proof-of-principle study is a novel avenue for drug delivery to the central nervous system.

David Fortin [4] in his paper entitled “Drug Delivery Technology to the CNS in the Treatment of Brain Tumors: The Sherbrooke Experience” also addresses challenges regarding drug delivery to the central nervous system and reviews strategies encompassing the path of the drug discovery from laboratory explorations to clinical applications. 

Waleed Mohammed-Saeid et al. [5], in their article entitled “ Inclusion Complexes of Melphalan with Gemini-Conjugated β-Cyclodextrin: Physicochemical Properties and Chemotherapeutic Efficacy in In-Vitro Tumor Models” report on how β-cyclodextrin (βCD) has been widely explored as an excipient for pharmaceuticals and nutraceuticals as it forms host–guest inclusion complexes and enhances the solubility of poorly soluble active agents. 

Asmita Poudel et al. [6], in their paper entitled “ Development and Characterization of Liposomal Formulations Containing Phytosterols Extracted from Canola Oil Deodorizer Distillate along with Tocopherols as Food Additives investigated formulation strategies for liposomes containing phytosterols obtained from canola oil deodorizer distillate, and tocopherols to overcome the challenges of thermo-sensitivity, lipophilicity and formulation-dependent efficacy of the nutraceuticals. The final aim is the development of functional foods, enriched with phytosterols and tocopherols. 

Jiahao Huang and colleagues [7], investigated the effect of phospholipids on a model compound, rosmarinic acid, and established relationship between membrane permeability and bioavailability on a dynamic gastrointestinal in vitro model, providing evidence for the complex interplay of these factors influencing bioaccessibility. 

Kevin Allen et al. [8] discuss highly reproducible method of determining its pharmacokinetics of antibodies for further pre-clinical development using 111-indium-labeled antibody in a melanoma tumour model, demonstrating superiority of this strategy compared to mass spectrometry. 

Hoda Soleymani Abyaneh et al. [9], in their paper entitled “Modulation of Hypoxia-Induced Chemoresistance to Polymeric Micellar Cisplatin: The Effect of Ligand Modification of Micellar Carrier Versus Inhibition of the Mediators of Drug Resistance” assessed strategies to overcome hypoxia-induced chemoresistance in a triple negative breast cancer cell line. They demonstrated that pharmacological inhibition of hypoxia significantly enhances cytotoxicity ofcisplatin encapsulated in in polymeric micelles. 

Zaid H Maayah et al. [10], reported that by chemically conjugating Vit-D to DOX the delivery of DOX into cancer cells increased and chemoresistance associated with DOX was mitigated via inhibition of survival pathways and induction of apoptosis.

Griffin Pauli et al. [11], discuss the advantages of solvent-assisted active loading technology (SALT) for liposomal encapsulation of compounds with low aqueous solubility. This new strategy is characterized by complete encapsulation, high loading efficiency and stable drug retention, leading to improvement of pharmacokinetic and pharmacodynamics parameters of the drugs. 

Farinaz Ketabat et al. [12], review treatment options in development for oral squamous cell carcinoma from new delivery systems to chronotherapy, and offer insight into future strategies in the field. 

Mahdi Roohnikan et al. [13], showcase research groups interested in the development of state-of-the-art transdermal delivery technologies. Within this short review, they aim to provide a critical overview of the development of these technologies in the Canadian environment. 

Esen Sokullu et al. [14], present an overview of applications of plant viruses and phages in drug discovery. Critical assessment of the status of virus-based materials in clinical research are summarized. The authors provide a critical assessment of challenges and opportunities presented by these highly stable and versatile delivery systems.

Bahman Homayun et al. [15], in their paper entitled “Challenges and Recent Progress in Oral Drug Delivery Systems for Biopharmaceuticals” outlines the advantages of oral drug delivery by reviewing the advantages and disadvantages different administration routes. Additionally mitigation strategies regarding challenges of each route are emphasized. 

Courtney Van Ballegooie et al. [16], depict physical strategies aimed towards release of drugs from liposomal formulation at their target site. The mechanism of drug release upon the use of energy sources, including ultrasound, magnetic fields, and external beam radiationis explained.

Ada W.Y. Leung et al. [17], provides a high-level review the most successful Canadian drug delivery systems translated to the clinic, leading to the formation of biotech companies. From the creation of research tools (Lipex Extruder and NanoAssemblr™) todevelopment of pharmaceutical products (Abelcet^®^, MyoCet^®^, Marqibo^®^, Vyxeos^®^, and Onpattro™) positive impacts on patients’ health are numerous. This review highlights the Canadian contribution to the development of these and other important liposomal technologies that have touched patients.

Grace Cuddihy et al. [18], in their paper entitled “The Development of Oral Amphotericin B to Treat Systemic Fungal and Parasitic Infections: Has the Myth Been Finally Realized?” discuss the development of an oral formulation of Amphotericin B to treat systemic fungal and parasitic infections. 

Taken together, these articles published in our special issue represents only a fraction of the drug delivery research and development ongoing within Canada but do serve as examples of the outstanding contributions Canadian’s have made to the discipline over the past 30 years. 
